# Delay in diagnosing a patient with mitochondrial encephalomyopathy, lactic acidosis, and stroke-like episodes (MELAS) syndrome who presented with status epilepticus and lactic acidosis: a case report

**DOI:** 10.1186/s13256-022-03613-2

**Published:** 2022-10-10

**Authors:** Ahmad F. Alenezi, Mariam A. Almelahi, Feten Fekih-Romdhana, Haitham A. Jahrami

**Affiliations:** 1 Ministry of Health, Kuwait City, Kuwait; 2 College of Medicine and Medical Sciences, Arabian Gulf University, Manama, Kingdom of Bahrain; 3 Department of Internal Medicine, Al-Amiri Hospital, Kuwait City, Kuwait; 4 Faculty of Medicine of Tunis, Tunis El Manar University, Tunis, Tunisia; 5 Department of Psychiatry “Ibn Omrane”, Razi Hospital, Manouba, Tunisia; 6 Ministry of Health, Manama, Kingdom of Bahrain; 7 Ministry of Health Building, Jamal Abdulnasser Street, Kuwait City, Kuwait

**Keywords:** MELAS syndrome, Mitochondrial encephalopathies, Stroke-like episode, Status epilepticus, Lactic acidosis

## Abstract

**Background:**

Mitochondrial encephalomyopathy, lactic acidosis, and stroke-like episode syndrome is a rare mitochondrial genetic disorder that can present with a variety of clinical manifestations, including stroke, hearing loss, seizures, and lactic acidosis. The most common genetic mutation associated with this syndrome is M.3243A>G. The main underlying mechanism of the disease relates to protein synthesis, energy depletion, and nitric oxide deficiency. Controlling disease complications and improving patient quality of life are the primary aims of treatment options.

**Case presentation:**

A 28-year-old Arabic female visited Al-Amiri Hospital in Kuwait. The patient was newly diagnosed with mitochondrial encephalomyopathy, lactic acidosis, and stroke-like episode syndrome following her admission as a case of status epilepticus requiring further investigation. The patient’s seizures were controlled, and she was evaluated to rule out the most serious complications by carrying out appropriate clinical, laboratory, and radiological imaging. The patient was discharged from the hospital after 2 weeks with a follow-up plan.

**Conclusion:**

This case report emphasizes the importance of considering mitochondrial encephalomyopathy, lactic acidosis, and stroke-like episode syndrome as a potential cause of status epilepticus with lactic acidosis in a young female patient with a past history of stroke-like episodes. It also stresses the most important workup to rule out every possible life-threatening complication to improve patients’ lives.

## Background

Mitochondrial encephalomyopathy, lactic acidosis, and stroke-like episodes are together known as MELAS syndrome [1, 2], a rare mitochondrial disease that is exclusively maternally inherited. The disease was first described in 1984 in a case report [3] by Dr. Steven Pavlakis and his colleagues. Although an accurate incidence was difficult to determine, the rate was estimated to be 0.2 per 100,000, for example, in Japan [4]. However, the carrier status rate was much higher, with a prevalence of 16.3 per 100,000 in Finland [5].

Different gene mutations [6–8] have been associated with MELAS syndrome. First discovered in 1990 [9, 10], the M.3243A>G mutation [11] in the mitochondrially encoded tRNA leucine 1 is also known as the *MT-TL1* gene, which was identified as the most common mutated gene linked to the disease. The main underlying mechanism of the disease is impairment of mitochondrial translation [12], which leads to a decline in protein synthesis and energy depletion, eventually resulting in mitochondrial dysfunction and an inability to generate an adequate amount of energy to support various organs with key roles in multiorgan dysfunction. On the other hand, recent studies have shown that nitric oxide (NO) deficiency [1, 13] is another part of the disease mechanism because it plays an important role in vascular smooth muscle relaxation. That is, NO deficiency may interfere with blood perfusion in the microcirculation of multiple organs, thereby resulting in dysfunction.

Moreover, the disease has a wide range of clinical presentations and involves different organ systems, such as the brain [14], muscles [2], heart [15], eye [16], cochlear system [17], thyroid [18], parathyroid gland [19], and adrenal glands [20]. Patients with MELAS syndrome can develop up to 31 different clinical symptoms [14, 21]; however, only seven cardinal symptoms are noted in more than 90% of patients. The symptoms include stroke, seizures, lactic acidosis, ragged-red fibers, exercise intolerance, and normal early development, and the age of onset is before 40 years.

Although seizures are one of the main symptoms of MELAS syndrome and are reported in more than 90% of cases [1], convulsive status epilepticus has not been fully reported in literature reviews as one of the clinical manifestations of MELAS syndrome, and its incidence and pathophysiology have not been studied. However, a few studies [22–24] have introduced status epilepticus as one of the manifestations of mitochondrial disorders.

In 2012, the MELAS study committee in Japan[4] published a new diagnostic criterion based on category A, that is, clinical findings of stroke-like episodes that include (1) headache with vomiting, (2) seizure, (3) hemiplegia, (4) cortical blindness or hemianopsia, and (5) acute focal lesions observed via brain imaging. Category B consists of evidence of mitochondrial dysfunction, such as (1) high lactate levels in plasma and/or cerebral spinal fluid or deficient mitochondrial-related enzyme activities, (2) mitochondrial abnormalities on muscle biopsy, and (3) definitive gene mutation related to MELAS syndrome. A definitive diagnosis of MELAS syndrome is assumed if two items from each category are present. However, a suspected diagnosis is considered when only one category A item and two category B items have been met.

Other common clinical manifestations include dementia and memory impairment [25], cortical vision loss [1], hearing impairment [26], and headache [1]. The estimated survival time based on a fully symptomatic patient is estimated to be between 10 and 16.9 years from the onset of focal neurological disease [27]. In terms of treatment options, no cure is available for MELAS syndrome, and all possible treatment methods aim to either control evolved complications (for example, using antiepileptic medications for seizures [28] and hearing aid devices for hearing loss [29]), improve quality of life, decrease crisis severity by minimizing the demand on mitochondria and maximizing their function, or enhance the production of NO by using l-arginine [30, 31], citrulline [31], coenzyme Q10 [32, 33], and riboflavin (B2) [34].

In light of the above discussion, this case report aims to highlight a rare genetic case first identified in Al-Amiri Hospital in Kuwait City, Kuwait. It also stresses the importance of obtaining a detailed history from patients and their families, which may elicit suspicion of a rare genetic disorder that may present with vague symptoms. This case report emphasizes the importance of considering MELAS syndrome as a potential cause of status epilepticus with lactic acidosis in a young female patient with a past history of stroke-like episodes. Additionally, it considers the most important workup to rule out every possible life-threatening complication to improve patients’ lives.

## Case history and presentation

A 28-year-old Arabic female who was known to have allergic rhinitis and bilateral hearing loss was admitted to Al-Amiri Hospital in Kuwait due to status epilepticus for further investigation on 14 June 2022. The patient’s symptoms started early in the morning on the admission day. Initially, she had sudden intermittent bilateral blurry vision when she presented to the Al-Bahar Eye Center, and she was discharged as no abnormalities were found on the eye examination and her blurry vision partially improved. However, later in the evening, the patient had another attack of blurry vision mainly in the right eye with photophobia, two episodes of vomiting with food content, and severe headache. The patient’s family decided to take her to the hospital emergency room (ER). En route to the hospital, the patient lost consciousness and developed tonic‒clonic seizures in the car that lasted for 90 seconds. The seizures aborted spontaneously; however, the patient did not gain consciousness for 30 minutes. Upon arrival at the Al-Amiri ER, the patient was unconscious with vital signs including a temperature of 37.1 °C, a heart rate of 112 beats per minute (bpm), a blood pressure of 172/93 mmHg, and an oxygen saturation of 98%. Five minutes later, the patient had another tonic‒clonic seizure and was treated with diazepam, levetiracetam, and midazolam. Consequently, the seizure was terminated.

A medical history was obtained from the patient’s mother. She had a normal developmental history, but her history also revealed gradual hearing loss in both ears when she was 24 years old. Therefore, she attended a specialized speech and hearing center and was diagnosed with sensorineural hearing loss, and she has used a hearing aid device since the diagnosis. At the age of 26, the patient had a stroke-like episode where she developed a headache, right arm and leg weakness, and slurred speech. The patient presented to a nongovernmental hospital where a brain computed tomography (CT) scan and magnetic resonance imaging (MRI) were performed. A neurologist suspected that she may have autoimmune encephalitis and therefore started corticosteroid treatment with a methylprednisolone injection and discharged her on oral prednisolone for 5 days. The patient’s condition improved gradually, but she did not attend the follow-up.

Nonetheless, the patient reported memory impairment at the age of 27 when she became forgetful and was unable to remember recent events and days. Another neurologist in a different nongovernmental hospital reviewed her previous records and suspected a mitochondrial disorder and thus recommended further genetic testing. The patient was transferred to the Kuwait Medical Genetic Center for genetic testing but decided not to follow-up. Her family history revealed that the patient’s maternal grandmother died at age 40 following a stroke. Additionally, a positive history of hearing loss was noted in three of the patients’ aunts. Her mother reported a mild degree of bilateral hearing loss and biting her tongue several times (lateral side) when she woke up. However, she was never admitted to the hospital for further evaluation. The patient was an unmarried female who was not sexually active and had no history of smoking, drugs or alcohol abuse. The patient’s height was 1.50 m, her weight was 33 kg, and her body mass index (BMI) was 14.7 kg/m^2^.

After status epilepticus resolved, a full physical examination was completed, with nonsignificant findings. Additionally, arterial blood gas was performed on admission, which showed metabolic acidosis (lactic acidosis) mixed with respiratory acidosis, with a blood pH of 7.19, pCO_2_ of 6.5 kPa, pO_2_ of 10.5 kPa, lactic acid of 11.4 mmol/L, and an HCO_3_ of 18.4 mmol/L. An urgent brain CT scan was also performed to rule out any acute central cause, and the results showed no evidence of acute brain insult. Given the recurrent seizures in a young female patient, meningoencephalitis needed to be ruled out. Therefore, a lumbar puncture was performed, and a cerebrospinal fluid (CSF) sample was taken for cell counting; protein, lactate, and glucose measurements; culture/sensitivity testing; and Gram staining (Table 1). Moreover, autoimmune screening was performed to rule out autoimmune encephalitis (Table 2). Additionally, blood samples were taken for a complete blood count (CBC), liver function tests (LFTs), and renal function tests (RFTs), which returned nonsignificant results.

**Table 1:** Cerebrospinal fluid testing

Test	Result	Reference range
RBCs	20 cells/cumm	0–10 cells/cumm
WBCs	5 cells/cumm	0–5 cells/cumm
Protein	1016 mg/L	150–450 mg/L
Glucose	3.2 mmol/L	2.2–3.9 mmol/L
Lactate	3.9 mmol/L	1.1–2.4 mmol/L
Gram stain	No pus cells	–
Culture and sensitivity	Sterile	–

*RBCs* Reb Blood Cells; *WBCs* White Blood Cells

**Table 2:** Autoimmune workup

Test	Result	Reference range
RF	20 IU/mL	0–20 IU/mL
C3	1.05 g/L	0.79–1.52 g/L
C4	0.30 g/L	0.16–0.38 g/L
Anti-thyroid peroxidase Ab	1004.5 IU/mL	0–75 IU/mL
Anti-ds DNA Ab (CLIA)	< 9.8 IU/mL	0–35 IU/mL
Anti-ds DNA Ab	Negative	Negative/Positive
Anti-CCP Ab	Negative	Negative/Positive
ANA	Negative	Negative/Positive
Anti-RNP/Sm	Negative	Negative/Positive
Anti-Sm	Negative	Negative/Positive
Anti-SS-A	Negative	Negative/Positive
Anti-SS-B	Negative	Negative/Positive
Anti-Scl-70	Negative	Negative/Positive
Anti-Jo-1	Negative	Negative/Positive
Anti-Centromere B	Negative	Negative/Positive
Anti-nucleosomes (NUC)	Negative	Negative/Positive
Anti-histone	Negative	Negative/Positive
Anti-ribosomal-P-protein	Negative	Negative/Positive
Anti-NMDAR	Negative	Negative/Positive
Anti-CASPR2	Negative	Negative/Positive
Anti-AMPAR1	Negative	Negative/Positive
Anti-LGI1	Negative	Negative/Positive
Anti-AMPAR2	Negative	Negative/Positive
Anti-GABARB1/B2	Negative	Negative/Positive

*RF* Rheumatoid Factor; *C3* complement 3; *C4* complement 4; *Anti-thyroid peroxidase Ab* anti-thyroid peroxidase antibody; *Anti-ds DNA Ab (CLIA)* anti-double stranded DNA antibody (Clinical Laboratory Improvement Amendments); *Anti-CCP Ab* anti-cyclic citrullinated peptide antibody; *ANA* anti-nuclear antibodies; *Anti-RNP/Sm* anti-nuclear ribonucleoprotein/Smith antibody; *Anti-Sm* anti-Smith antibody; *Anti-SS-A* anti-Sjögren's syndrome related antigen A antibody; *Anti-SS-B* anti-Sjögren's syndrome related antigen B antibody; *Anti-Scl-70* anti-scleroderma 70 antibody; *Anti-Jo-1* anti-histidy tRNA synthetase antibody; *Anti-NMDAR* anti-Anti-N-methyl D-aspartate receptor antibody; *Anti-CASPR2* anti-contactin associated protein-like receptor 2 antibody; *Anti-LGI1* anti-Leucine–Rich Glioma-Inactivated 1 antibody; *Anti-AMPAR2* anti-α-amino-3-hydroxy-5-methyl-4 isoxazolepropionic acid receptor 2 antibody; *Anti-GABARB1/B2* anti-gamma aminobutyric acid receptor B1/B2 antibody

**Fig. 1:**
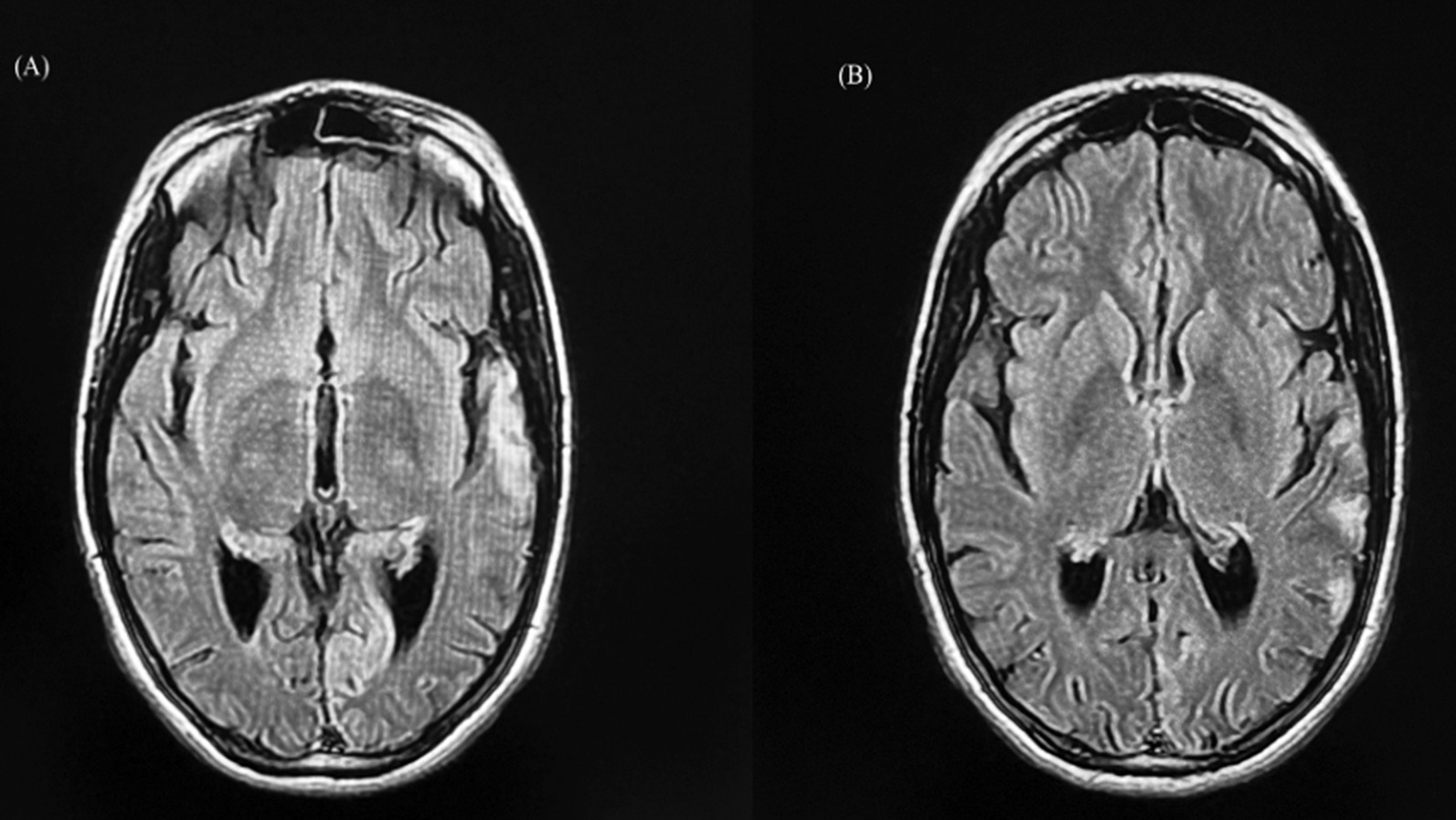
Brain MRI: (**A**) left temporal, parietal cortical and subcortical patchy areas of high signal intensity on T2 and Fluid attenuated inversion recovery Wigs (FLAIR WIGs). (**B**) left occipital and temporal patchy areas of high signal intensity

Because the lumbar puncture results ruled out an ongoing central nervous system (CNS) infection, an urgent brain MRI was performed, which showed left occipital, temporal, and parietal cortical and subcortical patchy areas of high signal intensity on T2 and Fluid attenuated inversion recovery Wigs (FLAIR WIGs) (Fig. 1). The radiologist’s impression was either status epilepticus-induced cerebral changes with subvolumic brain alterations due to the use of antiepileptic drugs or cerebral autosomal dominant arteriopathy with subcortical infarcts and leukoencephalopathy (CADSIL) syndrome with left-sided cerebral ischemic lesions. After consulting the neurology team and reviewing the patient’s previous reports, MELAS syndrome was suspected. The patient and her family were advised to undergo genetic testing for MELAS syndrome mutation, which they accepted. After contacting the Kuwait Medical Genetic Center, the authors received a previous report from the center regarding the patient, which showed that the patient had undergone genetic testing 8 months ago but did not follow up on the results. According to the results, the patient is a carrier of the m.3243A>G mutation in the *MT-TL1* gene in mitochondrial DNA, which is associated with MELAS syndrome. Following this discovery, a multidisciplinary team including internal medicine, neurology, genetics and metabolism, psychiatry, neuro-ophthalmology, and otorhinolaryngology clinicians met to discuss the current diagnosis, propose further management plans, and determine the prognosis.

Regarding the new-onset seizures, an electroencephalogram (EEG) was performed, which showed a slow background with intermittent diffuse slowing, consistent with a mild degree of diffuse encephalopathy, and left hemispheric dysrhythmia likely related to a lesion on that side, where some frontal slow waves had a brush-like appearance. The patient was started on levetiracetam, as it is a safe option for patients with mitochondrial disorders. Seizures were controlled, and the patient had no other attacks. The genetics and metabolism team recommended that the patient start supportive treatment for the MELAS syndrome crisis. The treatment included intravenous arginine 0.5 g/kg diluted in 500 mL of 5% dextrose infused over 2 hours, followed by the same dose infused over 24 hours for 3 days. This treatment was followed by oral arginine 2000 mg once, coenzyme Q10 30 mg three times a day (TDS), vitamin C 500 mg TDS, and riboflavin 100 TDS. However, due to the unavailability of intravenous arginine, we started the patient with the arginine formula available in the hospital, namely, a 2 g arginine sachet, with the rest of the medication.

Next, the multidisciplinary team decided to evaluate the patient for the most serious complication related to MELAS syndrome. First, to rule out abnormal cardiac conduction abnormalities, an electrocardiogram (ECG) and a 24-hour Holter study were performed, which showed a normal sinus rhythm with no arrhythmia. Second, regarding cardiomyopathy, an echocardiogram study was performed, and the report indicated concentric left ventricle remodeling with normal left ventricle systolic function, mild mitral regurgitation, and mild tricuspid regurgitation. For further evaluation, cardiac MRI was performed, which showed concentric mild left ventricular hypertrophy with no imaging signs of myocardial inflammatory or infiltrative changes.

Third, to rule out any abnormality associated with the endocrine system (that is, diabetes mellitus, abnormal thyroid or parathyroid function, and adrenal gland insufficiency), a hormone panel was sent (Table 3). Fourth, because the patient had intermittent blurry vision in the right eye, she was evaluated by a neuro-ophthalmologist. His findings were a normal fundus, slight disc pallor, and right homonymous hemianopia. A regular outpatient appointment was scheduled for follow-up. Fifth, the patient’s psychological and mental well-being was observed as she was extremely anxious, teary, devastated, and screaming in denial, especially after learning the news of her condition. Thus, further evaluation from the psychiatric team was requested. The psychiatric team assessed the patient’s case and reviewed her history. She was diagnosed with major depressive disorder (MDD) and generalized anxiety disorder (GAD). However, after establishing a regular psychiatric visit, the patient’s mental well-being improved, and she was scheduled for regular follow-ups as an outpatient to proceed with cognitive behavioral therapy. Sixth, in terms of fluctuating hearing loss and new-onset hyperacusis, audiometry was scheduled, and an outpatient appointment was set with the otorhinolaryngology department for further evaluation.

**Table 3:** Hormonal panel

Test	Result	Reference range
Cortisol	365 nmol/L	185–624 nmol/L
Aldosterone	950 pmol/L	190–830 pmol/L
Renin	17.40 ng/L	5.41–34.5 ng/L
Aldosterone/Renin ratio	54.60 pmol/ng	4.1–81.3 pmol/ng
TSH	0.580 ulU/mL	0.43–4.1 ulU/mL
PTH	3.30 pmol/L	1.3–9.3 pmol/L
HbA1c	5.2%	4.0–5.5% Nondiabetic

*TSH* thyroid stimulating hormone; *PTH* parathyroid hormone; *HbA1C* Hemoglobin A1C

After spending 2 weeks in the hospital and completing all the necessary investigations, the patient’s condition improved. Proper education was given to the patient and her family regarding the importance of regular follow-ups with each medical specialty involved in her case. Additionally, screening for carrier status in the patient’s mother and unaffected siblings was advised. The patient was discharged from the hospital with the same medication mentioned previously.

## Discussion

In the present case, the diagnosis of MELAS syndrome was not straightforward. Extensive work and team efforts were required to gather the necessary information to reach the diagnosis. Owing to the unusual presentation of the syndrome and the need for genetic testing to confirm the disease, a high index of suspicion was required. Finding a previous genetic testing report during this admission added considerable value to how we approached this condition. Given the nature of the disease and the presentation of status epilepticus, we did not expect a full recovery from the crisis. However, we aimed to control seizure attacks and carry out the necessary investigations to help prevent further complications of the disease. The patient was discharged from the hospital 2 weeks after admission. Her seizures were controlled, unlike the blurry vision in her right eye and migraine headaches, which were persistent. We set a follow-up plan for the patient with each special department that examined her during admission, such as internal medicine for general health and well-being, neurology for seizures and migraine headaches, otorhinolaryngology for hearing loss and new-onset hyperacusis, neuro-ophthamology for blurry vision, and psychiatry for MDD and GAD.

Indeed, the patient’s history showed a significant delay of more than 3 years in establishing the correct diagnosis. The patient’s misdiagnosis of autoimmune encephalitis when she had her first stroke-like episodes should be further investigated as she had multiple features suggestive of MELAS syndrome, but because the patient presented to a nongovernmental hospital, all records regarding that hospital admission were inaccessible. This delay may have contributed to the patient's recent crisis. Additionally, delayed provision of proper education regarding the patient’s genetic disease and the kind of manifestations expected in the future served as another source of delay. Similar to MELAS syndrome, various rare genetic and nongenetic disorders that may not have specific features or various clinical manifestations can be challenging to diagnose. From our recent experience with this case, we strongly suggest the establishment of a specialized unit designed to diagnose such challenging cases. In a small state such as Kuwait, establishing a unit in a highly advanced health center will improve general health practices, help diagnose rare cases, and allow the presentation of such cases to other hospitals and physicians who are more experienced and prepared for these cases in the future.

Another issue to consider is the nature of mitochondrial dysfunction in MELAS syndrome and its associations with multisystemic manifestations such as diabetes mellitus [2], hearing loss [29], and seizures [1]. Choosing certain medications to treat these manifestations may unmask the symptoms of MELAS syndrome and induce a MELAS crisis. Here, three common medications should be avoided, as they have negative impacts on the disease course, namely, valproic acid [35, 36], aminoglycosides [17], and metformin [37]. These medications are associated with mitochondrial toxicity, hearing loss, and lactic acidosis. Therefore, choosing appropriate medications for patients with MELAS syndrome is fundamental.

Accordingly, the main strength of this case report is the unusual presentation of MELAS syndrome, which was status epilepticus. The second strength is the multidisciplinary team and the close follow-up. By understanding the unique nature of the syndrome, each medical team investigated the possible complications of MELAS syndrome, ensuring early detection and treatment of any manifestation.

On the other hand, two limitations were observed. The first was the lack of an adult genetic and metabolic specialty, which led us to seek the help of the pediatric department. In addition, the number of pediatricians board-certified in genetics and metabolism is very low in Kuwait. The second limitation was the limited number of case reports and presentations due to the rarity of the disease. This research field (that is, case presentation and reporting) in the Kuwaiti health care system has not received much attention in recent years. A lack of skills resulted in limited knowledge about rare cases and hampered learning from others’ experiences.

## Conclusion

Reporting a rare case such as MELAS syndrome is important to help other physicians with minimal experience whenever they encounter such cases, and to provide them with guidance regarding proper treatment.

## Data Availability

Not applicable
